# Electrophysical Properties of the PMN–PT–PS Solid Solution

**DOI:** 10.3390/ma11081292

**Published:** 2018-07-26

**Authors:** Dariusz Bochenek, Ryszard Skulski, Przemysław Niemiec

**Affiliations:** Faculty of Computer Science and Material Science, Institute of Technology and Mechatronics, University of Silesia in Katowice, 41-200 Sosnowiec, Poland; ryszard.skulski@us.edu.pl (R.S.); przemyslaw.niemiec@us.edu.pl (P.N.)

**Keywords:** PMN–PT, ferroelectric materials, relaxor materials, sol–gel, hysteresis loop

## Abstract

The (1 − *y*) ((1 − *x*)Pb(Mg_1/3_Nb_2/3_)O_3_–*x*PbTiO_3_)–*y*PbSnO_3_ solid solution (PMN–PT–PS) was obtained and investigated in the present paper. For the analysis of the influence of the PbSnO_3_ component on the electrophysical parameters, the compositions from the rhombohedral phase, tetragonal phase, and a mixture of these phases were selected. The six compositions of the PMN–PT have been obtained using sol–gel methods (for values of *x* equal to 0.25, 0.28, 0.31, 0.34, 0.37, and 0.40). The ceramic samples of the 0.9(PMN–PT)–0.1(PS) solid solution have been obtained using the conventional ceramic route. X-ray diffraction (XRD), energy dispersive spectrometry (EDS), and microstructure measurements were performed, as well as tests regarding the dielectric, ferroelectric, piezoelectric properties and electric conductivity of the PMN–PT–PS ceramic samples versus temperature. Results of the measurements show that the obtained PMN–PT–PS materials have good electrophysical properties and are well suited for use in micromechatronic and microelectronic applications.

## 1. Introduction

The lead-based (1 − *x*)Pb(Mg_1/3_Nb_2/3_)O_3_–*x*PbTiO_3_ (PMN–PT) solid solution belongs to the ferroelectric/relaxor family with perovskite structure [1]. The phase diagram of PMN–PT has been investigated by several authors [2,3,4,5,6,7]. Depending on the content of PbTiO_3_ in the composition, the PMN–PT solid solution has a rhombohedral structure (for *x* < 0.31), a tetragonal structure (for *x* > 0.37), or shows a mixture of tetragonal and rhombohedral phases (morphotropic phase boundary, MPB) for *x* in the range (0.31< *x* < 0.37). Recently, the MPB has been considered a region in which the low symmetry phases, i.e., monoclinic or orthorhombic, exist [3,8,9]. The PMN–PT material exhibits a low thermal expansion behavior up to the Curie temperature (*T_m_*), and shows a broad and frequency dependent diffuse phase transition that is characteristic of relaxor materials [10,11,12]. Good piezoelectric and electrostrictive properties of the PMN–PT solid solutions allow the use thereof in broad applications. The PMN–PT materials are well suited for use as actuators in smart structure systems that require precise and highly reproducible displacements. For *x* ≤ 0.1, the PMN–PT compositions are used for actuator applications involving large voltage-induced displacements [13,14]. PMN–PT compositions with a higher PT amount *x* (especially with *x* from the range of 0.30 to 0.35—i.e., from the morphotropic area) found usage in piezoelectric transducer and sensor applications [15,16,17,18].

In the present paper, the (1 − *y*) ((1 − *x*)Pb(Mg_1/3_Nb_2/3_)O_3_–*x*PbTiO_3_)–*y*PbSnO_3_ solid solution (PMN–PT–PS) was obtained and investigated. The introduction of PbSnO_3_ to the based PMN–PT composition gives an additional possibility to influence the temperature-dependent parameters and obtain the material with optimal parameters. The six compositions of the PMN–PT were obtained using sol–gel methods (for *x*: 0.25, 0.28, 0.31, 0.34, 0.37, 0.40). PMN–PT/PS samples were designed in proportions of 90/10. The introduction of an additional component into the base PMN–PT composition with the presence of tin was intended to reduce the width of the hysteresis loop while maintaining optimal electrophysical parameters, as well the polarizability of the tin cation (in common valence state at the level of 2.83 Å^3^), possible candidates for B position in the perovskite-type structure, a similar ionic radius of occupied cations, and a lack of toxicity. The sol–gel method enables obtaining a ceramic powder with optimal parameters at a low synthesis temperature, which preserves the stoichiometric composition [11,19,20].

## 2. Experimental

### 2.1. Preparation of the Ceramic Materials

In the present paper, the six compounds of the 0.9((1 − *x*)PbMg_1/3_Nb_2/3_O_3_–*x*PbTiO_3_)–0.1PbSnO_3_ (PMN–PT–PS) solid solutions (where values of *x* are from 0.25 to 0.40) were obtained using sol–gel methods, and are next investigated. The chemical compositions were the following:

(i). 0.9(0.75 PbMg_1/3_Nb_2/3_O_3_–0.25 PbTiO_3_)–0.1 PbSnO_3_ (PPP0.25),
(ii). 0.9(0.72 PbMg_1/3_Nb_2/3_O_3_–0.28 PbTiO_3_)–0.1 PbSnO_3_ (PPP0.28),
(iii). 0.9(0.69 PbMg_1/3_Nb_2/3_O_3_–0.31 PbTiO_3_)–0.1 PbSnO_3_ (PPP0.31),
(iv). 0.9(0.66 PbMg_1/3_Nb_2/3_O_3_–0.34 PbTiO_3_)–0.1 PbSnO_3_ (PPP0.34),
(v). 0.9(0.63 PbMg_1/3_Nb_2/3_O_3_–0.37 PbTiO_3_)–0.1 PbSnO_3_ (PPP0.37),
(vi). 0.9(0.60 PbMg_1/3_Nb_2/3_O_3_–0.40 PbTiO_3_)–0.1 PbSnO_3_ (PPP0.40).

The liquid PMN solutions were obtained as a result of the reaction between Nb(OC_2_H_5_)_5_ (99.95% Sigma–Aldrich, Steinheim, Germany) and Mg(OC_2_H_5_)_2_ (95%, Fluka, Buchs, Germany), and successively as a result of addition lead acetate (II), while the liquid PT solutions were obtained as a result of the reaction between Pb(CH_3_COO)_2_ (99.99%, POCH, Gliwice, Poland) and Ti(CH_3_CH_2_CH_2_O)_4_ (98%, Sigma–Aldrich, Germany, Steinheim). In the following step, the PMN–PT compounds were obtained from the mixture of liquid PMN and liquid PT in a proper proportion. A detailed description of the sol–gel technology of the obtaining of PMN–PT powders have been described in the previous work [11]. After drying the gels, the obtained powder was burned at 550 °C/h to remove organic parts.

The PbSnO_3_ (PS) powder was obtained as a result of the reaction between lead oxide PbO (99.99%, POCH, Gliwice, Poland) with excess 3.5 mol.% and tin oxide SnO_2_ (99.9% Aldrich, St. Louis, MO, USA). Input oxides were milled in a ball mill (Fritsch, Pulverisette 6, Idar-Oberstein, Germany) in ethyl alcohol for 15 h. Next, the powder was calcined at 850 °C for 4 h.

The PMN–PT and PS powders were mixed in ball mill (in ethyl alcohol, for 15 h), in a proportion of 90/10, respectively. The densification of the ceramic samples was carried out by the free sintering method at conditions of 1250 °C for 3 h [19]. After sintering, the ceramic samples were ground, polished, and annealed, and silver paste electrodes were put on both of the surfaces.

### 2.2. Characterization

The X-ray measurements at room temperature were performed using a (PANalytical, Phillips X’Pert Pro, Eindhoven, The Netherlands) diffractometer (Cu–Kα radiation). The data were collected in the 2*θ* range from 10° to 60°, in steps of 0.02 degrees, with an integration time of 4 s/step. Microstructure, EDS (energy dispersive spectrometry) tests were carried out using a Field Emission Scanning Electron Microscope (Jeol Ltd., JEOL JSM–7100 TTL LV, Tokyo, Japan). Prior to the SEM/EDS analyses, the samples were coated with gold to provide electrical conductivity and avoid any charging effects. The dielectric measurements were performed using an LCR meter (QuadTech, Inc., 1920 Precision LCR meter, Maynard, MA, USA) during a heating cycle (in temperature range from 20 °C to 320 °C) at frequencies of the measurement field from 0.1 kHz to 1.0 MHz. DC electrical conductivity has been measured using an electrometer (Keithley Instruments, Inc., 6517B, Cleveland, OH, USA) within the temperature range from 20 °C to 320 °C. Dielectric hysteresis loops *P*-*E* were investigated using a Sawyer–Tower circuit and a high voltage amplifier (Matsusada Precision Inc., HEOPS–5B6, Kusatsu, Japan) in the temperature range from room temperature to 130 °C. The data were stored on a computer disc using an A/D, D/A transducer card (National Instruments) and LabView computer program. Electromechanical measurements were carried out using an optical displacement meter (Philtec Inc., Annapolis, MD, USA, D63) and a high-voltage amplifier (HEOPS-5B6) at room temperature.

## 3. Results and Discussion

**Figure 1:**
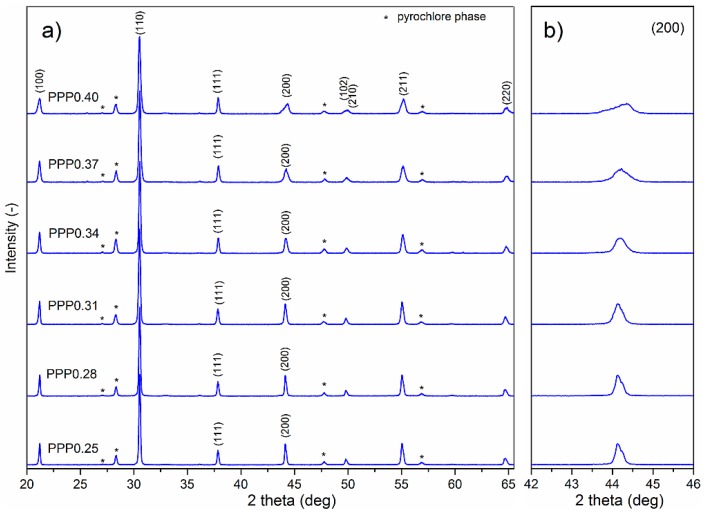
(**a**) XRD patterns for (1 − *y*) ((1 − *x*)Pb(Mg_1/3_Nb_2/3_)O_3_–*x*PbTiO_3_)–*y*PbSnO_3_ (PMN–PT–PS) solid solutions; (**b**) selected enlarged region; * pyrochlore phase.

The XRD patterns of PMN–PT–PS powders at room temperature are shown in Figure 1a. The XRD tests show that the obtained materials, beside the perovskite phase, exhibited also a small amount of the pyrochlore phase (Pb_2_Nb_2_O_7_). The amount of the perovskite phase in the obtained PMN–PT–PS materials was calculated from the following equation [21]:

(1) $$P_{phase}=\left(\frac{I_{perov}}{I_{perov}+I_{pyroch}}\right)\times 100\%,\;$$

where *I_perov_* is the intensity of the highest perovskite peak (110) and *I_pyroch_* is the intensity of the highest pyrochlore peak (222). The highest amount of the pyrochlore phase is observed in tetragonal PMN–PT–PS samples and in the samples from the morphotropic (MPB) area (Table 1).

Analyzing the XRD patterns in Figure 1, the changes in the shape of the (200) line of the perovskite phase with the increase in content of PbTiO_3_ in the PMN–PT component of the PMN–PT–PS solid solutions can be seen. Starting from *x* = 0.28 (for the PMN–PT rhombohedral phase), the pattern of (220) has a single maximum (R3*m* space group exists) and for *x* = 0.40 (PMN–PT from tetragonal phase), the (200) maximum consists of two parts (originating from the P*m* and P4*mm* space groups). In the case of the PMN–PT from the MPB area (for *x* = 0.34), the small reflections can be divided into more components (coexistence of R3*m*, C*m*, P*m*, and P4*mm* space groups) characteristic for monoclinic phases [11,22]. The changed shape of the diffraction (200) peak with the increase PT is presented in Figure 1b. From the selected enlarged region, it is seen that the (200) peaks become flattened and broad. The XRD tests also reveal that the influence of the PS component in PMN–PT–PS solid solutions is imperceptible, in comparison with Ref. [11]; however, an increased amount of the pyrochlore phase is observed. 

**Figure 2:**
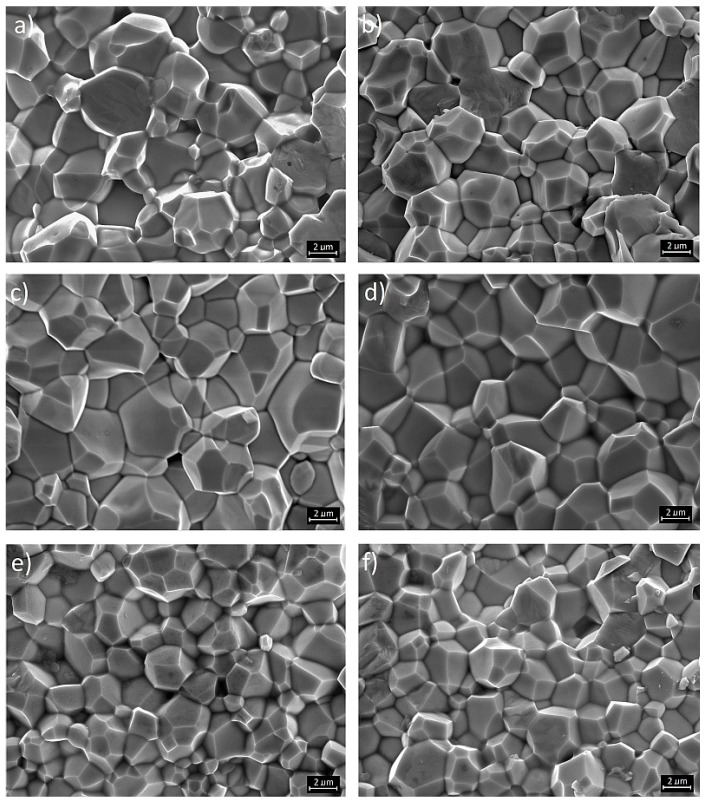
SEM images of PMN–PT–PS solid solutions: (**a**) PPP0.25; (**b**) PPP0.28; (**c**) PPP0.31; (**d**) PPP0.34; (**e**) PPP0.37; and (**f**) PPP0.40.

For SEM measurements, the ceramic samples were fractured, and on the examined surfaces (area of the fractured samples), a thin layer of gold was spread. The microstructures of the PMN–PT–PS materials are characterized by high density with regular and well-crystallized grains and clearly visible grain boundaries (Figure 2), without visible grains coming from the pyrochlore phase, as shown in Ref. [23]. For the obtained compositions, the breakthrough occurs at the grain boundaries, which proves the high mechanical strength of the grains. In the case of the PPP0.25 sample, cracking through the grain (to a lesser extent) is also observed (there is a mixed nature of cracks). The microstructure of the PPP0.25 and PPP0.28 compositions shows high grain homogeneity with the average grain size *r* = 4–5 μm.

For higher contents of PbTiO_3_ in a solid solution of PMN–PT–PS (PPP0.31 and PPP0.34 compositions from the morphotropic area), the heterogeneity of the microstructure grains increases, and there is an increase in the average grain size. In the case of the PPP0.37 and PPP0.40 compositions, the microstructure becomes fine-grained. However, this is accompanied by the increase of the heterogeneity of the microstructure (there are both small and very large grains). The heterogeneity of the microstructure increases when there are both small and large grains in the ceramic materials. The research showed no clear trend of change in the grain size of the PMN–PT–PS compositions, depending on the amount of PT. This is the effect of the different relations between the electrophysical properties and the crystalline structure in the tetragonal as well as in rhombohedral phases. It is well-known that the crystal structure and microstructure of ceramic materials are affected by the accurate and properly carried out technological process of obtaining the ceramic samples. On the other hand, the crystal structure and microstructure affect the electrophysical parameters of the ceramics. 

**Figure 3:**
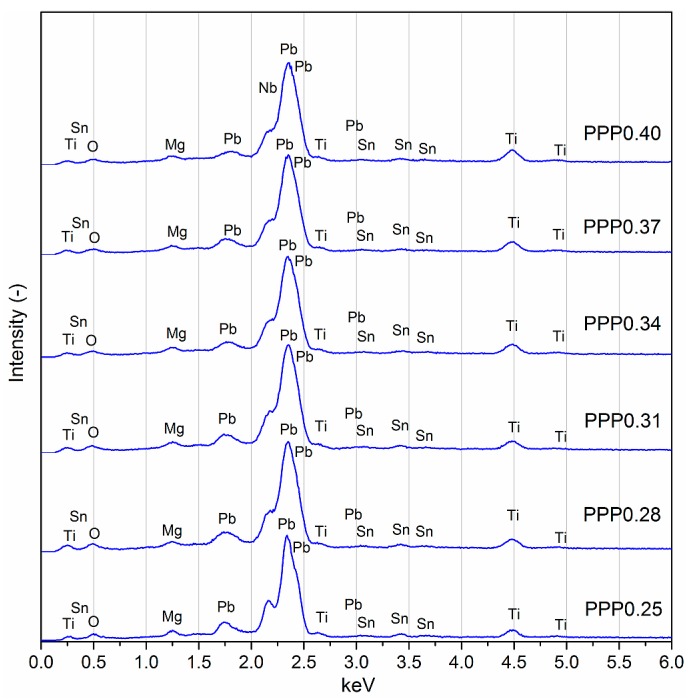
Energy dispersive spectrometry (EDS) tests of the PMN–PT–PS solid solutions.

Homogeneity studies on the composition of solid solutions of PMN–PT–PS were carried out using the EDS tests (Figure 3). The EDS measurements (based on point and surface analysis) confirmed the qualitative composition of the obtained samples without the presence of foreign elements. In Table 2, the percentage of the individual components of the PMN–PT–PS compositions are presented. In the graph, the increase of the amount of lead and titanium in the PMN–PT–PS composition manifests in the increase of intensities of the respective peaks. In the case of all of the samples, lead, magnesium, and tin deficiency and a small titanium and niobium excess are observed, as compared with theoretical calculations. All of the presented deviations from the initial composition are within the acceptable range.

**Figure 4:**
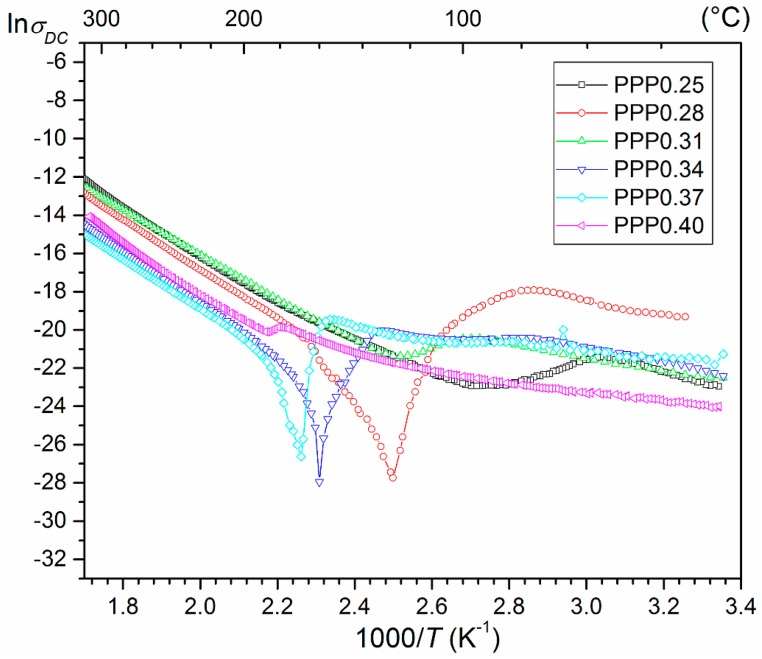
The ln*σ_DC_*(1/*T*) relationship for the PMN–PT–PS solid solutions.

At room temperature, the *ρ_DC_* resistivity of the PPP ceramic samples is in the range from 1.2 × 10^9^ Ωm to 1.2 × 10^9^ Ωm. The temperature dependencies of ln*σ*_DC_(*T*) for the investigated PPP compositions are presented in Figure 4. For all of the compositions (excluding PPP0.40), just below the phase transition temperature, a sharp decrease of electrical conductivity is observed, and there is a maximum above the phase transition temperature (PTCR i.e., the effect of the positive temperature coefficient of resistivity). The minima of ln*σ*_DC_(*T*) are the most visible for compositions PPP0.28, PPP0.34, and PPP0.37. Due to its creation at high temperature, both the defects as well as vacancies, and significant differences in conductivity at lower and high temperatures are observed. As the temperature rises, the reduction of grain boundary resistance causes a reduction of the mobility barrier of load carriers participating in the grain boundary conductivity.

**Figure 5:**
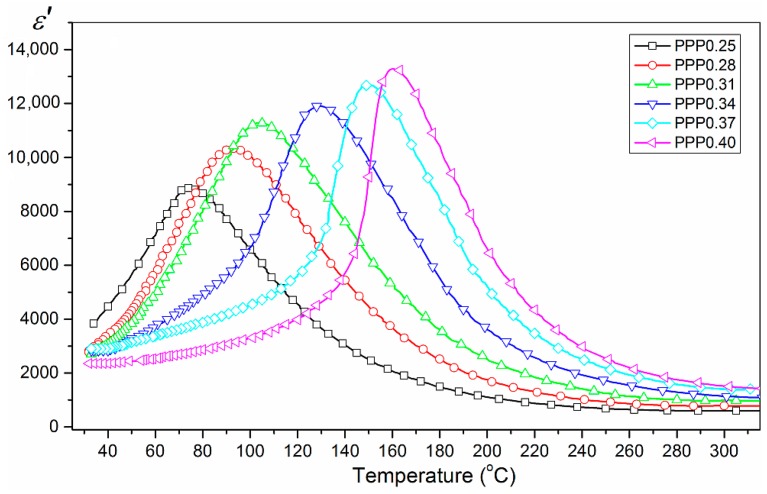
Dependencies *ε’*(*T*) for PMN–PT–PS ceramics (cooling cycle for 1 kHz).

The results of temperature investigations of dielectric permittivity *ε*(*T*) measured at 1.0 kHz are presented in Figure 5. The PMN–PT–PS samples exhibit a broad peak of dielectric permittivity *ε*(*T*) which is often referred to as a diffuse phase transition. The broader temperature range of *T_m_* exists for samples with smaller *x* (the ferroelectric–paraelectric phase transition takes place in a wider temperature range). The temperature investigations of dielectric permittivity tests also reveal that the increase of the PT component in the PMN–PT–PS solid solutions (with a constant volume of PS component) caused an increase of the values of the maximum of dielectric permittivity at temperature *T_m_*. At the same time, the shift of temperature in which phase transition occurs toward higher temperatures is visible. Simultaneously, the greater amount of PT in PMN–PT–PS solid solutions reduces the width diffuse of the phase transitions (the ferroelectric–paraelectric phase transition takes place in a more narrow temperature range). Measurements of dielectric permittivity show that, along with the increase in the PT content, there are three clearly visible tendencies of the results: (i) a *T_m_* increase, (ii) a *ε_max_* at *T_m_* increase, and (iii) a decrease in the diffusion of the phase transition. 

**Figure 6:**
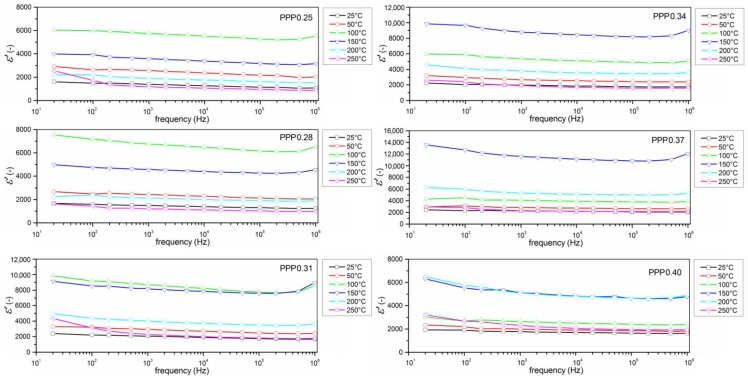
Frequency dependence of the real part of dielectric permittivity for PMN–PT–PS ceramics with various *x* at different temperatures.

Figure 6 shows the frequency dependence of the real part of dielectric permittivity *ε’* of the PPP samples, measured in temperature range from 25 °C to 250 °C. Figure 6a–f shows that all of the ceramic samples have a similar dielectric behavior, depending on the frequency of the measuring field, and they all have a Debye-like relaxation. *ε’*(*f*) plots displays a step decrease at the frequency.

**Figure 7:**
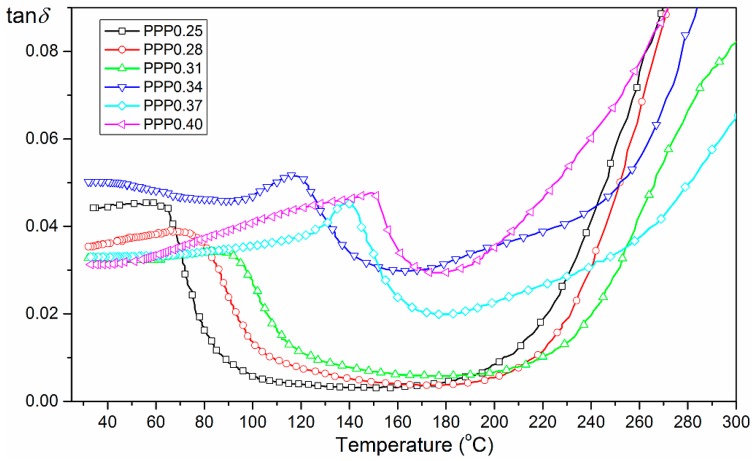
Temperature dependencies tan*δ*(*T*) for PMN–PT–PS ceramics (cooling cycle for 1 kHz).

The results of temperature investigation of dielectric loss for PMN–PT–PS samples are presented in Figure 7. The measurements show very low values of tan*δ*(*T*) of the PMN–PT–PS samples. For all of the compositions, the increase in temperature from room temperature to *T_m_* temperature causes a slight increase in the value of dielectric loss. The dielectric loss is reduced next just before the phase transition. Just above the *T_m_* temperature, there is a temperature range in which the PMN–PT–PS solid solution shows the lowest values of dielectric loss. In case of the PPP0.25 composition, this area is the widest, while in the PPP0.40 composition, it is the narrowest. A further increase in temperature causes a rapid increase in dielectric loss associated with an increase in electrical conductivity of the ceramic materials. Similar results of dielectric tests are presented in Ref. [24]. The obtained results of temperature investigations of dielectric loss correlate well with the electrical conductivity measurements (Figure 4).

**Figure 8:**
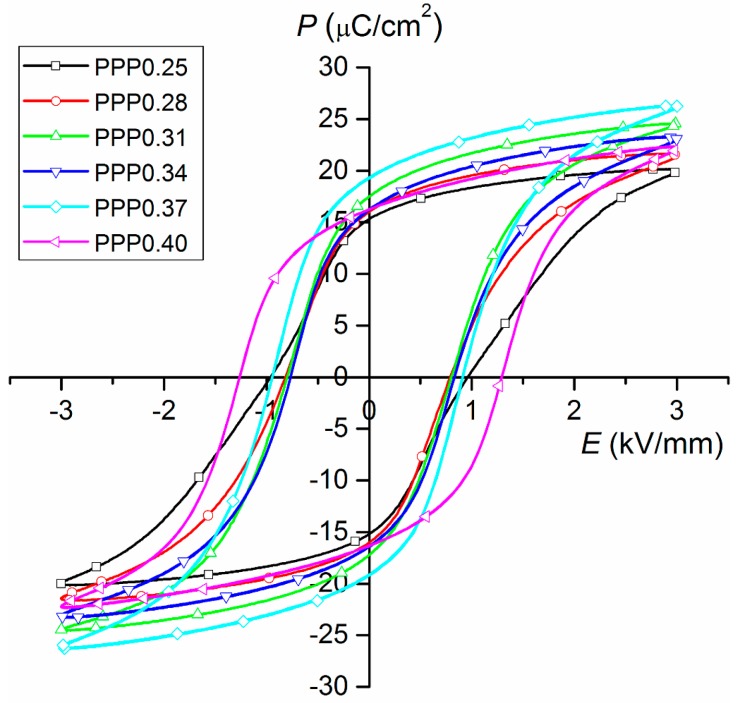
Hysteresis loops for PMN–PT–PS samples at room temperature (frequency 1 Hz).

**Figure 9:**
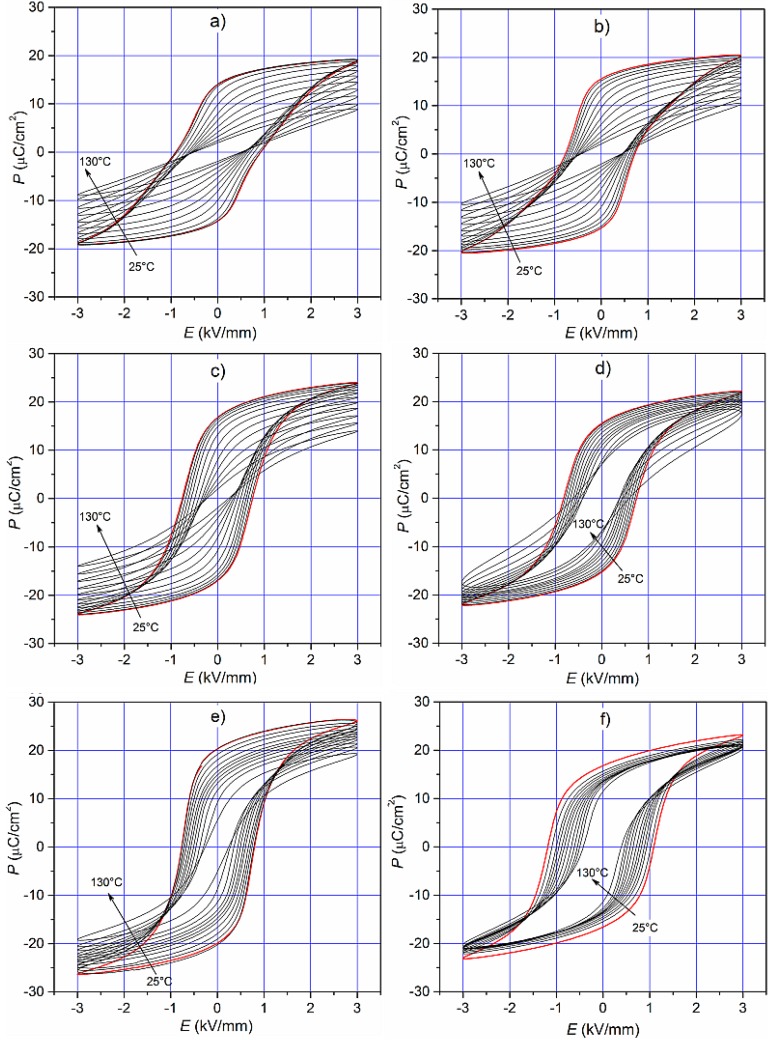
The hysteresis loops for PMN–PT–PS samples at various temperatures: (**a**) PPP0.25; (**b**) PPP0.28; (**c**) PPP0.31, (**d**) PPP0.34; (**e**) PPP0.37; and (**f**) PPP0.40 (frequency 1 Hz).

This cycling of the electric field leading to polarization switching is an essential property of ferroelectrics, and it also gives rise to the formation of the ferroelectric hysteresis loop (in most cases, its presence provides an experimental confirmation for the switchable polarization and underlying ferroelectric nature) [25]. The hysteresis loops for PMN–PT–PS solid solutions obtained at room temperature are presented in Figure 8. For all of the obtained compositions, at electric field 3.0 kV/cm, the *P*-*E* hysteresis loops exhibits saturation. The hysteresis loop width (i.e., coercive field) depends on the content of the PT component in the PMN–PT–PS solid solution. At room temperature, the lowest value of the coercive field is found for the PPP0.28 sample (composition from the rhombohedral phase), while the largest one has the PPP0.40 sample (composition from the tetragonal phase). Compared to the unmodified PMN–PT materials presented in Ref. [11] for PMN–PT–PS materials, the *P*-*E* loops are narrower. In the case of PMN–PT [11], the application of higher electric fields (above 2 kV/mm) initiated the electrical breakthrough of the samples. An additional PS element introduced into the PMN–PT provided the possibility of applying a higher electric field to all of the samples (in our case, 3 kV/mm), also at high temperatures (Figure 9).

The temperature dependencies *P*(*E*) in temperature range of 25 °C to 130 °C (for 1 Hz) of the PMN–PT–PS solid solutions are presented in Figure 9. With increasing temperature, the spontaneous polarization decreases, and the hysteresis loop becomes less saturated. For the PPP0.25, PPP0.28, and PPP0.31 compositions of the above phase transition, the hysteresis loops of the loops have a standard shape (characteristic paraelectric loops with small amounts of loss). Due to the high temperatures of the phase transition, the compositions from the tetragonal phase (PPP0.37 and PPP0.40) retain high saturation of the *P*-*E* loops at high temperatures. One exception to the above regularities is the PPP0.34 composition, the loops of which at higher temperatures begin to be characteristic of materials with losses (characteristic “lossy” round loops).

The rectangularity coefficient of the hysteresis loop (at room temperature) was calculated from the following Formula (2):

(2) $$\;a_{rec}=\frac{P_{R}}{P_{max}}\;$$

where *a_rec_*—rectangularity coefficient, *P_R_*—remnant polarization, and *P_max_*—maximum value of polarization. The rectangularity coefficient *a_rec_* of the PMN–PT–PS solid solution (Table 1) is the highest for samples with rhombohedral structure and the lowest for samples from the MPB area.

**Figure 10:**
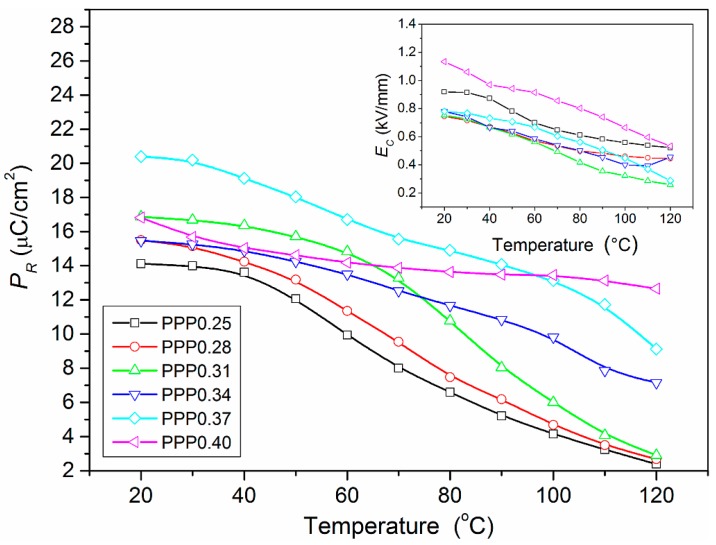
The temperature dependencies of remnant polarization *P_R_* and coercive filed *E_C_* (inside) for PMN–PT–PS solid solutions.

The temperature dependencies *P_R_*(*T*) and *E_C_*(*T*) in the temperature range of 25 °C to 120 °C for all of the PMN–PT–PS solid solutions are presented in Figure 10. For all of the samples, with the temperature rise, to reach temperatures close to *T_m_*, there is a trend of decreasing *P_R_* and *E_C_* parameters. At higher temperatures, an increase in *P_R_* and *E_C_* are observed (the loop loses saturation and takes the characteristic form of materials with losses).

**Figure 11:**
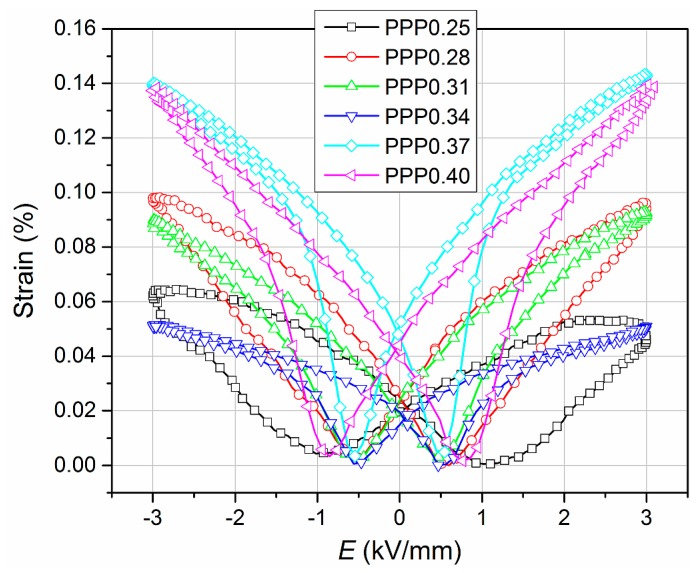
Strain vs. electric field of the PMN–PT–PS ceramics (1 Hz).

In an ideal defect-free single crystal material that is poled perfectly, the remnant strain is represented by its lattice distortion [26]. As is well known, the electric field induces strain in the ceramic sample, which is caused by the domain switching, the number of polarization states, electrostriction, and the applied electric field [27]. Figure 11 shows the results of electromechanical investigations (*S*-*E*) at room temperature for all of the unpolarized investigated ceramics using a frequency of 1 Hz. The PMN–PT–PS ceramic samples exhibit an appreciable *S*-*E* behavior. The change in the character of strain mechanism may be seen with increasing titanium content. In the samples with high titanium content, the strain versus the electric field is typical for piezoelectric materials (linear *S*-*E* dependency). The values of the remnant strain are 0.020%, 0.024%, 0.021%, 0.017%, 0.049%, and 0.042% for the PPP0.25, PPP0.28, PPP0.31, PPP0.34, PPP0.37, and PPP0.40 samples, respectively. Higher values of the remnant strain occur for the tetragonal phase compositions, which is characteristic of materials with a perovskite structure. In the case of the PPP0.34 sample, both the shape of the loop and the value of the remnant strain differ significantly from the other samples. This can be attributed to the accumulation of various reasons, such as the randomness of grain orientations that naturally confine the orientation of domains, depolarization fields arising from defects, pinning of domains, etc. [26].

Compared to the unmodified PMN–PT materials presented in previous work [11] for PMN–PT–PS materials, the *S*-*E* loops are narrower (for samples with the same PT content, except for the PPP0.25 composition). Furthermore, the *S*-*E* loops obtained for PMN–PT–PS materials do not show a pronounced asymmetry, as in the case of the PMN-PT materials [11] and another one [28].

The large signal piezoelectric coefficient $d_{33}^{*}$ was calculated from following formula:

(3) $$d_{33}^{*}=\left(\frac{S_{max}}{E_{max}}\right)\;$$

where $d_{33}^{*}$—piezoelectric coefficient, *S_max_*—maximum value of mechanical strain, and *E_max_*—maximum value of electric field. The $d_{33}^{*}$ piezoelectric coefficient of the PMN–PT–PS solid solutions was calculated as a maximal value of the derivative ∂*S*/∂*E* obtained from the *S*-*E* electromechanic loops. The piezoelectric coefficient was measured under a 3 kV/mm electric field and a frequency of 1 Hz for all samples, which was summarized in Table 1.

## 4. Conclusions

The six compositions from the rhombohedral, tetragonal and mixture of these phases of the (1 − *y*)((1 − *x*)Pb(Mg_1/3_Nb_2/3_)O_3_–*x*PbTiO_3_)–*y*PbSnO_3_ solid solution (PMN–PT–PS) were obtained successfully.

The XRD tests show that the obtained materials have a perovskite structure with a small amount of the pyrochlore phase. The microstructures of the PMN–PT–PS materials are characterized by high density with regular and well-crystallized grains with clearly visible grain boundaries. The temperature measurements of the dielectric properties of the PMN–PT–PS samples show a diffuse phase transition. The increase of the PT component in PMN–PT–PS solid solutions (with a constant volume for the PS component) caused an increase in the maximum values of dielectric permittivity at temperature *T_m_* and a reduction in the width of diffusion of the phase transition region (i.e., the ferroelectric–paraelectric phase transition takes place in a narrower temperature range). The PMN–PT–PS solid solutions have very low values of dielectric loss. The hysteresis loop width (coercive field) depends on the content of the PT component in the PMN–PT–PS solid solution. At room temperature, the lowest value of the coercive field is found for the PPP0.28 sample (composition from the rhombohedral phase), while the largest one has the PPP0.40 sample (composition from the tetragonal phase).

The research proved that the introduction of an additional component into the base PMN–PT composition, with the presence of tin, improves the sinterability of the ceramic samples, and reduces the width of the *P*-*E* and *S*-*E* loops in comparison with PMN–PT materials (undoped PS component) [11].

The presented measurements show the PMN–PT–PS materials with good electrophysical properties are well suited for use in micromechatronic and microelectronic applications as actuators in smart structure systems that require precise and highly reproducible displacements.

## Figures and Tables

**Table 1:** The electrophysical parameters of the PMN–PT–PS solid solutions.

Parameter	PPP0.25	PPP0.28	PPP0.31	PPP0.34	PPP0.37	PPP0.40
*P_phase_* (%)	90.58	91.04	90.80	86.54	89.37	88.82
*T_m_* (°C) ^a^	74	93	104	129	150	161
*ε_r_* ^a^	3870	2770	2670	2825	2905	2370
*ε_max_* ^a^	8860	10,333	11,275	11,902	12,695	13,270
tan*δ* at *T_r_*^a^	0.044	0.035	0.033	0.050	0.033	0.031
tan*δ* at *T_m_*^a^	0.025	0.020	0.021	0.042	0.034	0.033
*E_C_* (kV/mm) ^b^	0.919	0.745	0.753	0.781	0.78	1.133
*P_R_* (μC/cm) ^b^	14.11	15.50	16.86	15.46	20.39	16.81
*P_s_* (μC/cm) ^b^	18.83	19.14	21.40	20.64	22.31	18.07
*a_rec_*	0.76	0.74	0.71	0.70	0.74	0.73
$d_{33}^{*}$ (pm/V) ^c^	153.5	313.5	295.2	169.5	476.2	446.3

^a^ for 1 kHz cooling cycle, ^b^ for 1 Hz, ^c^ calculated from Formula (3) for *E_max_* = 3 kV/mm, *T_r_*—room temperature.

**Table 2:** Theoretical and experimental percentages of elements (expressed as oxides) of the PMN–PT–PS ceramics.

Oxide Formula	PPP0.25		PPP0.28		PPP0.31		PPP0.34		PPP0.37		PPP0.40	
	th. (%)	ex. (%)	th. (%)	ex. (%)	th. (%)	ex. (%)	th. (%)	ex. (%)	th. (%)	ex. (%)	th. (%)	ex. (%)
PbO	66.60	65.26	66.48	63.99	66.37	63.75	66.25	63.92	66.14	63.17	66.03	62.90
MgO	2.64	2.42	2.52	2.36	2.41	2.28	2.29	2.45	2.18	2.09	2.06	2.15
Nb_2_O_5_	20.56	22.53	20.14	22.96	19.73	22.30	19.33	22.41	18.93	22.01	18.53	21.99
TiO_2_	5.81	5.93	6.48	6.61	7.14	7.53	7.80	8.19	8.45	8.88	9.09	9.27
SnO_2_	4.39	3.85	4.37	4.08	4.35	4.14	4.33	3.98	4.31	3.84	4.29	3.69

th.—theoretical calculation, ex.—experimental results.
